# Distribution of Rotundone and Possible Translocation of Related Compounds Amongst Grapevine Tissues in *Vitis vinifera* L. cv. Shiraz

**DOI:** 10.3389/fpls.2016.00859

**Published:** 2016-06-21

**Authors:** Pangzhen Zhang, Sigfredo Fuentes, Yueying Wang, Rui Deng, Mark Krstic, Markus Herderich, Edward W. R. Barlow, Kate Howell

**Affiliations:** ^1^Faculty of Veterinary and Agricultural Sciences, University of Melbourne, ParkvilleVIC, Australia; ^2^Australian Wine Research Institute, MooroolbarkVIC, Australia; ^3^Australian Wine Research Institute, UrrbraeSA, Australia; ^4^UMR 1083 Sciences pour l’Oenologie, Institut National de la Recherche AgronomiqueMontpellier, France

**Keywords:** rotundone, sesquiterpenes, secondary metabolite, phloem translocation, herbivore attack, pepper aroma

## Abstract

Rotundone is an attractive wine aroma compound, especially important for cool climate Shiraz. Its presence in wine is mainly from the grape skin, but can also be found in non-grape tissues, such as leaves and stems. Whether rotundone is produced independently within different grapevine tissues or transported amongst non-grape tissues and grape berries remains unclear. The current study investigated the distribution of this compound in different vine tissues during development and studied the most likely mode of rotundone translocation—via phloem—using stable isotope feeding. In addition, local production of rotundone induced by herbivore feeding was assessed. Results showed that rotundone was firstly detected in the petioles and peduncles/rachises within the development of *Vitis vinifera* L. cv. Shiraz. Different grapevine tissues had a similar pattern of rotundone production at different grape developmental stages. In the individual vine shoots, non-grape tissues contained higher concentrations and amounts of rotundone compared to berries, which showed that non-grape tissues were the larger pool of rotundone within the plant. This study confirmed the local production of rotundone in individual tissues and ruled out the possibility of phloem translocation of rotundone between different tissues. In addition, other terpenes, including one monoterpenoid (geraniol) and six sesquiterpenes (clovene, α-ylangene, β-copaene, α-muurolene, δ-cadinene, and *cis*/*trans*-calamenene) were, for the first time, detected in the ethylenediaminetetraacetic acid-facilitated petiole phloem exudates, with their originality unconfirmed. Unlike other herbivore-induced terpenes, herbivorous activity had limited influences on the concentration of rotundone in grapevine leaves.

## Introduction

Rotundone has been reported to be the main compound responsible for the “peppery” aroma in Shiraz grapes and wine, and is the first sesquiterpene compound that have been shown to contribute to grape and wine aroma (Siebert et al., 2008; Wood et al., 2008). Rotundone is derived from its sesquiterpene precursor, α-guaiene, via aerial oxidation and biosynthetic transformation (Huang et al., 2014; Drew et al., 2015; Takase et al., 2016; Zhang et al., 2016). Within grape berries, rotundone is only produced in berry skin (exocarp), and its concentration levels vary amongst different Shiraz clones (Herderich et al., 2012, 2013; Matarese et al., 2013). Rotundone is extracted into the juice through the crushing process and, ultimately, to the wine during fermentation (Herderich et al., 2012, 2013). Rotundone is also present at high levels in grapevine leaves and stems, therefore, when these organs are added to the fermentation process, rotundone concentration in wine can be increased up to sixfold compared to fermenting just berries (above 200 ng l^-1^; Capone et al., 2012). Production of rotundone in other grapevine tissues, such as petioles, rachises, and peduncles, has yet to be investigated. Whether rotundone is transported between the source and sink tissues or it is produced independently in different plant tissues remain unclear. Previous studies observed higher concentrations of rotundone in the berries produced by high-vigor grapevines, in seasons with high water availability, and in grape bunches located closer to leaves (Scarlett et al., 2014; Herderich et al., 2015; Zhang et al., 2015a,b), which could be related to higher shade and lower temperatures or a potential relocation of rotundone from the vegetative organs to grape berries. On the other hand, herbivore-induced sesquiterpene production does not happen systemically within the whole plant in response to leaf feeding by herbivores, but only at the wounding leaf site and distal leaf parts (Köllner et al., 2013), which indicates the localized formation of sesquiterpenes.

In vascular plants, the phloem can transport nutrients, defense compounds, chemical signals, and a number of secondary metabolites from source to sink tissues (Turgeon and Wolf, 2009), and it is the most likely pathway for a secondary metabolite like rotundone to be transported among plant tissues. Previous studies have shown that two monoterpene derivatives were transported via the phloem in *Asarina scandens* (Scrophulariaceae) and *Catalpa speciosa* (Northern Catalpa; Gowan et al., 1995; Turgeon and Medville, 2004). Conversely, monoterpene glycosides and their precursors were not transported into berries from other parts of the vine in Muscat grapevines unless an active transport mechanism for a specific compound was present (Gholami et al., 1995). To the best of our knowledge, transportation of sesquiterpene compounds in phloem has not been studied in any plant, although Hampel et al. (2005) did demonstrate that sesquiterpenes could be biosynthesized in the phloem of carrot roots. Nevertheless, the current research investigates the most likely phloem transportation pathway to rule out or confirm the possibility of rotundone translocation in grapevine.

Rotundone belongs to the terpene chemical family, many of which are considered herbivore-induced plant volatiles (Arimura et al., 2005; Howe and Jander, 2008). It is possible that rotundone or its precursors are produced locally in specific tissues as defensive compounds to protect grapevines from herbivores (Zakir et al., 2013). The two main impacts of herbivorous insects on plants are physical damage and oral elicitor secretions (Howe and Jander, 2008). Previous studies reported elevated leaf monoterpenes and sesquiterpenes concentration in simulations of herbivore physical damage to plant leaves (Mithöfer et al., 2005; Bricchi et al., 2010). Phytohormones, including salicylic acid (SA), jasmonic acid (JA), methyl jasmonate (MeJA), ethylene and its precursor 1-aminocyclopropane-1-carboxylic acid (ACC), played important roles in regulating plant responses to herbivore elicitors by activating or suppressing terpenoid biosynthesis genes (Arimura et al., 2005; Howe and Jander, 2008; Bari and Jones, 2009; Gómez-Plaza et al., 2012; Pieterse et al., 2012). Therefore, phytohormones can be used as surrogate of herbivore elicitors when assessing grapevine terpene synthesis. This research investigates local rotundone production in leaves under herbivorous feeding and simulated damage and chemical treatment conditions to confirm whether local rotundone production could be regulated by herbivore activity.

Here, we fully examine the distribution of rotundone amongst grapevine tissues at different grape developmental stages, and study the absolute amount of rotundone reserves in individual grapevine tissues. We further investigate the two possibility of a source–link relationship among grapevine tissues from: (i) the transportation of rotundone, α-guaiene, and other sesquiterpenes via phloem; (ii) the local production of rotundone induced by herbivores and mimic damage/chemical treatments. Results showed that rotundone is widely distributed in all vegetative tissues of grapevines, and it was shown for the first time the concentration of rotundone in petioles, peduncles/rachises, and canes. Non-grape tissues contained higher concentrations and total amounts of rotundone compared to grape berries at both veraison and harvest. The current study also confirmed that rotundone was most likely produced independently by different tissues, while its transportation via the phloem is unlikely. However, other sesquiterpenes have been observed in the phloem exudate using ethylenediaminetetraacetic acid (EDTA)-facilitated exudation method, and the originality of these compounds requires further investigation. Additionally, herbivorous activity had limited impacts on local rotundone production in grapevine leaves, and rotundone was unlikely to be a major component of herbivore-induced grapevine volatiles.

## Materials and Methods

### Chemicals

**FIGURE 1:**
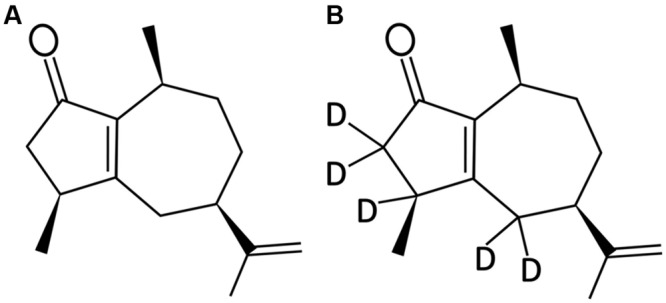
**Chemical structure of **(A)** (-)-rotundone and **(B)** d5-rotundone**.

Rotundone ((3S,5R,8S)-3,4,5,6,7,8-hexahydro-3,8-dimethyl-5-(prop-1-en-2-yl)-1(2H)-azulenone; **Figure 1A**) and ^2^H_5_-rotundone (**Figure 1B**) were synthesized as previously described (Siebert et al., 2008; Wood et al., 2008). A reference standard of α-copaene was purchased from Sigma-Aldrich (Castile Hill, NSW, Australia) for phloem sesquiterpene analysis. Working solutions of standards were prepared volumetrically in ethanol and stored at 4°C until required. Analytical-grade potassium L-tartrate monobasic, tartaric acid, SA, JA, MeJA, ACC, and other chemicals were also obtained from Sigma-Aldrich. HPLC-grade solvents for rotundone extraction, including ethyl acetate, *n*-pentane, methanol, and ethanol, were supplied by Rowe Scientific (Doveton, VIC, Australia). Water was purified using the Milli-Q system (Millipore Australia, Bayswater, VIC, Australia).

### Vineyard Trials: Distribution of Rotundone in Grapevines

Distribution of rotundone among grapevine tissues was investigated in a commercial vineyard (The Old Block, Mount Langi Ghiran 37.31°S, 143.15°E) located in the Grampians wine region of Victoria, Australia. The vineyard is located approximately 15.5 km east of the nearest Bureau of Meteorology (BOM) weather station at Ararat Prison (Australian BOM Station No. 089085). At this weather station, the weather data of the two studied growing seasons (2012–2013 and 2013–2014) were recorded and shown in Supplementary Table 1. The vineyard was planted in 1968 with *Vitis vinifera*, cv. Shiraz on its own roots at 3.0 m between rows and 1.8 m between vines (1852 plants ha^-1^), with rows oriented northeast to southwest. Grapevines are trained to a vertical shoot positioned trellis, and irrigated by a drip-irrigation system when required at a rate of 5.76 l h^-1^ vine^-1^). No significant pest or disease pressure was observed during the experimental seasons and management was performed following the company’s guidelines.

#### Experiment A1

**FIGURE 2:**
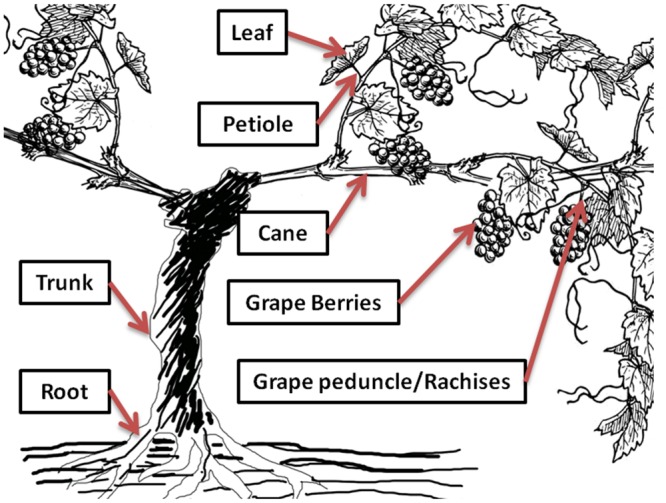
**Distribution of rotundone in different grapevine tissues.** Selected grape and non-grape tissues were analyzed for rotundone concentration at specific phenological stages of grapevine growth.

Green grapevine tissues (**Figure 2**), including grape bunches with peduncles/rachises and leaves with petioles were sampled in triplicate across the whole vineyard block (*n* > 30 grapevines for each sampling date) at fortnightly intervals from pea-size berry (E-L 31, January 9, 2014) until the beginning of commercial harvest (E-L 38, April 8, 2014) for the 2013–2014 growing season. Phenological stages of grapevine were identified based on the E-L system (Coombe and Iland, 2004). Non-green tissues, such as trunk, cane, and root were not sampled in this experiment. The collected samples were separated into grape berries, peduncles/rachises, leaves, and petioles, packed in zip-lock plastics bags, and stored at -20°C before analysis.

#### Experiment A2

Ten vines from a single row of the experimental vineyard were selected to study the rotundone distribution in individual vine shoots, since large within-vineyard variations in berry rotundone concentration was previously reported for this experimental block (Scarlett et al., 2014; Zhang et al., 2015a). The selected vines were all from in the high rotundone concentration region of the experimental block (Zhang et al., 2015a). The vines were selected on the basis of uniformity of canopy and cluster development. Selected vines were separated by at least two vines as buffer. One representative shoot from each selected vine were collected at around 80% veraison (E-L 35, February 28, 2014) and at the beginning of commercial harvest (E-L 38, April 8, 2014) in the 2013–2014 growing season, as the period from veraison to harvest has been identified as the key stage of rotundone development (Geffroy et al., 2014; Zhang et al., 2015b). The sampled shoots were separated into grape berries, peduncles/rachises, leaves, petioles, and canes (**Figure 2**), weighed, packed in zip-lock plastics bags, and stored at -20°C before analysis.

### Glasshouse Trials

Two-year-old *V. vinifera* L. cv. Shiraz grapevines on their own roots were used to assess the possible translocation and local production of rotundone. Plants were grown in 20 l pots and maintained at 20°C in a glasshouse (System Garden, Parkville, The University of Melbourne, Australia). An automatic drip irrigation system was installed with a set irrigation volume of 0.85 l vine^-1^ day^-1^. A commercial NPK fertilizer (Osmocote, Scotts Australia Pty Ltd, Bella Vista, NSW, Australia) was used to provide nutrients. No significant pest or disease pressure was observed during the experimental period.

#### Assessment of Rotundone Transportation with Stable Isotope Feeding

##### Experiment B1

Two experimental groups were prepared with three potted vines per treatment as replicates. Control group grapevines had no contact with the d_5_-rotundone solution. From each treatment vine, 15 randomly selected, fully expanded, still attached, and mature leaves were partially dipped in 10 ml aqueous d_5_-rotundone solution (12.9 ng ml^-1^) without any surfactant in plastic zip-lock bags. d_5_-Rotundone solutions were applied 1 week prior to berry harvest, and contact was avoid with other leaves from the grapevines. Treated leaves with petioles, untreated leaves with petioles, and grape bunches from each grapevine were sampled after 7 days. Treated leaves and petioles were rinsed with Milli-Q water to remove any residual d_5_-rotundone solution from the surface of tissues. Grape leaves and petioles were separated at sampling and stored in separate zip-lock bags. For grape bunches, grape berries and grape peduncles/rachises were separated after sampling and stored in separate zip-lock bags. Leaf and petiole samples were collected from each control vine on the same day. All samples were stored at -20°C until d_5_-rotundone identification analysis.

##### Experiment B2

Three experimental groups were prepared with three potted vines per treatment as replicates. Control vines had no contact with d_5_-rotundone solution (control-phloem). For the “whole leaves” treatment, 15 whole, fully expanded, still attached, and mature leaves from each treatment vine were placed in contact with 10 ml aqueous d_5_-rotundone solution (25.8 ng ml^-1^) in plastic zip-lock bags. For the “cut leaves” treatment, 15 fully expanded, still attached, and mature leaves from each treatment vine were cut in half under Milli-Q water then dipped into 10 ml aqueous d_5_-rotundone solution (25.8 ng ml^-1^) in plastic zip-lock sandwich bags. Leaf samples were collected 4 days after treatments applied and immediately transferred to the laboratory for phloem exudate extraction as described below.

Phloem exudate extraction was performed using the EDTA method (Gowan et al., 1995). For this purpose, 15 leaves were cut at the petiole base, immediately re-cut under Milli-Q water to avoid cavitation, and subsequently rinsed to avoid contamination of the cellular fluid. The leaves were then placed in a 10 ml plastic screw cap tube, and the base of petioles were immersed in 0.2 ml of a 20 mM EDTA in 20 mM piperazine-N,N′-bis(ethanesulfonic acid) (PIPES) buffer (pH 6.8) solution. The leaves were then removed from the petiole. The tubes were capped and placed in a humidified chamber in complete darkness at 22°C for 8 h. The solutions were then prepared for gas chromatography-mass spectrometry (GC-MS) identification of d_5_-rotundone and rotundone. Phloem exudate samples were prepared for each treatment replicate. Five control-phloem samples were prepared: 100 μl d_5_-rotundone (516 ng ml^-1^ in ethanol) was added to the first as “positive control-phloem,” no d_5_-rotundone to the second as “negative control-phloem,” and the remainder were used for sesquiterpene analysis.

#### Assessment of Local Rotundone Production Induced by Herbivore, Phytohormones, and Physical Wounding

##### Experiment C1

*Epiphyas postvittana* larvae were prepared following the published protocol (Singh et al., 1985). Briefly, *E. postvittana* eggs collected from adult moths were placed in plastic cups and maintained in a growth chamber under controlled conditions (21 ± 2°C, 50 ± 5% relative humidity, 14 h light and 10 h dark) until larvae emerged. Three experimental groups were prepared with six potted vines per treatment as replicates. Around 20 leaves from each vine were treated with: (i) no *E. postvittana* larvae treatment (control [Ctrl]); (ii) low-density *E. postvittana* larvae treatment (LD, larvae number: 1 per leaf); (iii) high-density *E. postvittana* larvae treatment (HD, larvae number: 6 per leaf). In the two larval treatment groups, 7-day-old larvae were gently tapped onto vine foliage between 4 and 5 pm, and larvae were allowed to feed for 7 days. After removal of the larvae, vine leaves were harvested and stored at -20°C until subsequent analysis. In the LD and HD treatment groups, leaves were separated in to left and right half abaxial sides with the petiole at the bottom, and larvae were only allowed to feed on the left half. The damage left half and undamaged right half of the leaves were separated, and the damaged half were labeled “LD-T” or “HD-T,” while the undamaged half were labeled “LD-C” and “HD-C.”

##### Experiment C2

Seven experimental groups were conducted with six potted vines per treatment as replicates, and experimental groups were separated by plastic film to prevent interactions. All chemicals used in the experiment were prepared in deionized Milli-Q water and stored at 4°C until required: (i) control (Milli-Q water); (ii) ACC (1 mM); (iii) ACC + JA (1 mM + 1 mM); (iv) JA (1 mM); (v) JA + SA (1 mM + 1 mM); (vi) SA (1 mM); and (vii) MeJA (1 mM). For each treatment, 10 ml of the liquid was evenly sprayed on all leaves of each vine three times on the 1st, 4th, and 7th day of treatment. Treated leaves were sampled in zip-lock plastic bags on the 8th day of the experiment and stored at -20°C until analysis.

##### Experiment C3

Four experimental groups were conducted with six potted vines per treatment as replicates, and experimental groups were separated with plastic film to prevent interactions. Mechanical wounding was only conducted on fully expanded leaves: (i) no physical damage (control); (ii) each leaf on the vine randomly punched 10–15 times per day with a 1 mm diameter hole puncher (1 mm LD); (iii) each leaf on the vine randomly punched 30–45 times per day with a 1 mm diameter hole puncher (1 mm HD); (iv) each leaf on the vine randomly punched 10–15 times per day with 5 mm diameter hole puncher (5 mm LD). Treated leaves were sampled in zip-lock plastic bags on day 7 and stored at -20°C until analysis.

### SPME-GC-MS Analysis of Rotundone and Sesquiterpenes

#### Quantification of Rotundone

Grape berries were prepared for rotundone quantification based on a published protocol (Siebert et al., 2008; Geffroy et al., 2014). Non-grape materials samples were also prepared for rotundone quantification based on a previously published protocol (Wood et al., 2008) with the following modification: 3 g of finely cut up grape peduncles/rachises, leaves, petioles, and canes samples were soaked in mixture of 24 ml ethanol, 24 ml Milli-Q water, and 100 μl of d_5_-rotundone (516 ng ml^-1^ in ethanol as internal standard) for 72 h at 22°C, then topped up to 200 ml with Milli-Q water and filtered prior to solid-phase extraction (SPE) as previous protocol description (Siebert et al., 2008). SPE residue supernatant was air dried with nitrogen and reconstituted in 0.5 ml ethanol and 9 ml Milli-Q water. Samples were analyzed in the Australian Wine Research Institute (AWRI, Adelaide, SA, Australia) using a published solid-phase microextraction-GC-MS (SPME-GC-MS) protocol (Geffroy et al., 2014). The target ions used were *m/z* 223 with 208 as qualifier for d_5_-rotundone, *m/z* 218 with *m/z* 203 for rotundone (Siebert et al., 2008).

#### Identification of d5-Rotundone

Stable isotope feeding treatment samples were prepared for d_5_-rotundone identification based on the same rotundone quantification protocol (Siebert et al., 2008; Wood et al., 2008; Geffroy et al., 2014) with the following modification: no d5-rotundone was not added to samples as internal standard during sample preparation process, except for the “positive control” sample of experiment B1, where 100 μl d_5_-rotundone (516 ng ml^-1^ in ethanol) were added. Phloem exudates samples were prepared for d_5_-rotundone identification following a similar protocol with the following modifications: the phloem exudates in EDTA and PIPES buffer solution were topped up to 100 ml with Milli-Q water and then subjected to SPE, air dry, and SPME-GC-MS analyses as above.

Grape petiole phloem exudates were prepared for sesquiterpene analysis following established protocols (Parker et al., 2007; Zhang et al., 2016; Zhang et al., unpublished manuscript). Phloem exudates were transferred into an headspace (HS)-SPME vial (Agilent Technologies, Santa Clara, CA, USA; 20 ml) and mixed with 2 ml saturated brine and 500 μl α-copaene (200.64 μg l^-1^ in ethanol) as internal standard and shaken for 24 h at 22°C. Samples were analyzed by SPME-GC-MS at the AWRI following published protocols (Parker et al., 2007; Zhang et al., 2016; Zhang et al., unpublished manuscript).

### Statistical Analysis

The rotundone concentration in different grapevine tissues at different ripening stages were compared using one-way analysis of variance (ANOVA; *p* < 0.05) in the CoStat software (veraison 6.4, CoHort Software, Monterey, CA, USA) to analyze statistical differences of mean values. Grapevine leaf rotundone concentrations in herbivore, physical wounding, and phytohormone treatment groups were also compared using one-way ANOVA (*p* < 0.05). The percentage of physical damage on physical wounding leaf samples was analyzed using customized code written in MatLab^®^ veraison 2014a (The MathWorks, Inc., Natick, MA, USA). Pearson’s test was used to assess correlations between grapevine leaf rotundone concentrations and physical damage (CoStat). A *p*-value < 0.05 was considered statistically significant.

## Results and Discussion

### Distribution of Rotundone in Grapevines

**FIGURE 3:**
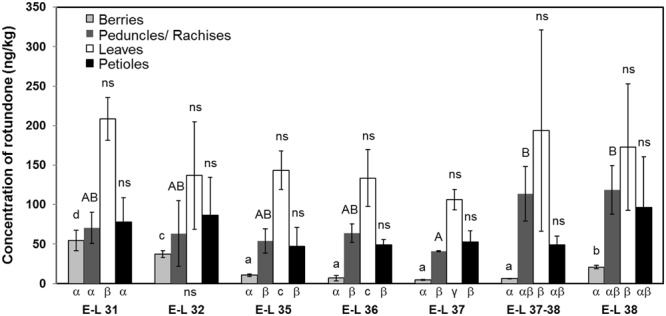
**Comparison of rotundone concentration among non-grape materials and berries at different grapevine phenological stages in the 2013–2014 growing season.** The grape materials were collected in the vineyard, and were the average of the area. a, b, c, d and A, B were used to label significant differences (*p* < 0.05) among different phenological stages, while α, β, γ were used to label significant differences (*p* < 0.05) among different grapevine materials. Standard deviation of each measurement was labeled on each bar.

The distribution of rotundone was firstly investigated at vineyard-wide scale, and the rotundone concentrations in individual vine tissue were analyzed at different phenological stages (**Figure 3**). Overall, the highest concentrations of rotundone in berries, leaves, petioles, and peduncles/rachises were observed at E-L 31 (54.6 ± 12.9 ng/kg), E-L 31 (208.6 ± 27.1 ng/kg), E-L 38 (96.5 ± 64.1 ng/kg), and E-L 38 (118.6 ± 30.7 ng/kg), respectively. The lowest concentrations of rotundone is berries, leaves, petioles, and peduncles/rachises were observed at E-L 37 (4.8 ± 0.7 ng/kg), E-L 37 (106.4 ± 13.0 ng/kg), E-L 35 (47.1 ± 24.3 ng/kg), and E-L 37 (40.9 ± 0.5 ng/kg), respectively. Large deviation in rotundone concentration were observed in grape and non-grape tissue samples, which was consistent with the previous report of large within-vineyard variations in rotundone concentration (Zhang et al., 2015a). As a result, statistical differences in rotundone could hardly be established among the same type of tissue from different phenological stages. Nevertheless, rotundone in all tissues tend to reach the minimum concentrations at phenological stages near veraison (E-L 35 to E-L 37). In addition, result showed a similar rotundone distribution pattern amongst grapevine tissues across the whole ripening period (E-L 31 to E-L 38) with the highest/lowest concentration of rotundone consistently found in leaves and berries, respectively. Importantly, this study has reported for the first time the detection of rotundone at high concentrations in petioles and peduncles/rachises. This knowledge is especially important for wineries conducing whole bunch fermentation, as significant amount of peduncles and rachises are included in the primary fermentation process, where rotundone is extracted into juice/wine (Herderich et al., 2013).

**FIGURE 4:**
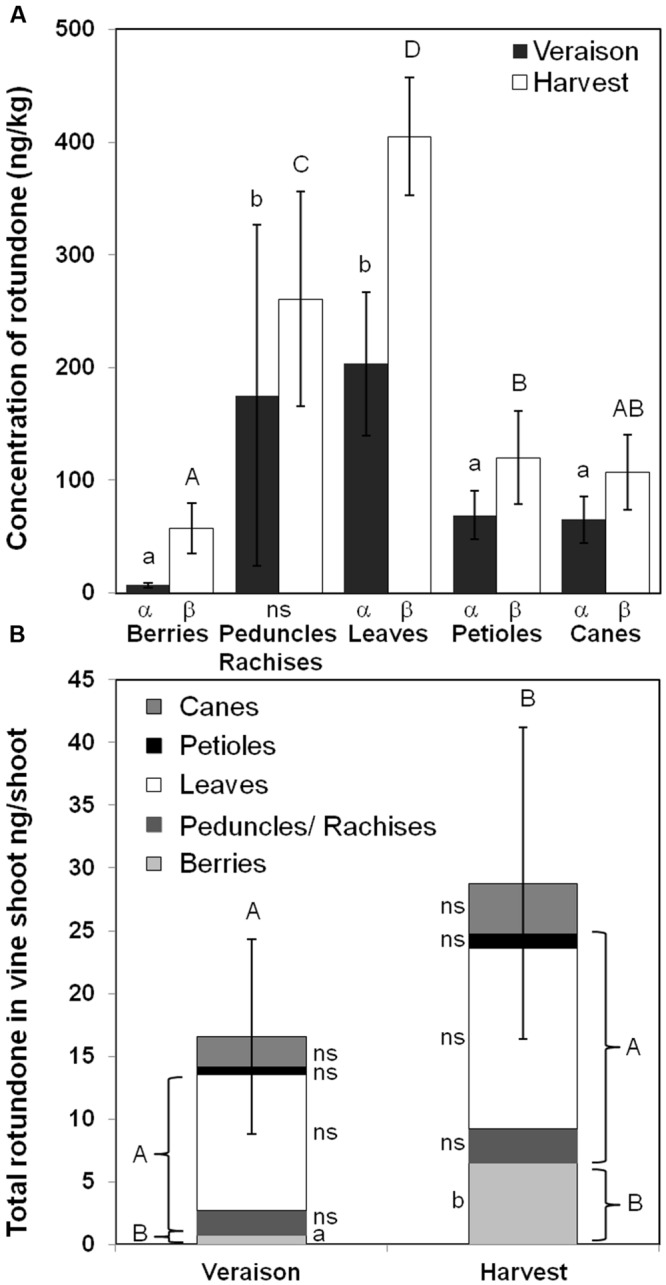
**Comparison of rotundone production in non-grape materials and berries at a specific vineyard location in the 2013–2014 growing season. (A)** Concentration of rotundone among different non-grape materials and berries at veraison and harvest, where a, b, c, d and A, B, were used to label significant differences (*p* < 0.05) among grapevine materials, while α, β, γ were used to label significant differences (*p* < 0.05) between veraison and harvest. Standard deviation of each measurement was labeled on each bar. **(B)** Comparison of total rotundone content in individual vine shoot between veraison and harvest, where a, b were used to label the significant differences (*p* < 0.05) between veraison and harvest, A, B were used to label the significant differences (*p* < 0.05) between total rotundone content in leaves, petiole, and peduncles/rachises and that of berries. Standard deviations of total rotundone content at veraison and harvest were labeled on each bar.

To minimize the influences of within-vineyard variation in rotundone and better understand rotundone distribution, individual shoot from selected vines in the high rotundone region of the vineyard were analyzed at veraison and harvest (**Figure 4**). As expected, higher concentrations of rotundone were observed in all tissues compared to the vineyard scale experiment (Zhang et al., 2015a). Significant increases in the concentration of rotundone in berries, leaves, petioles, and shoots were observed at harvest compared to veraison (**Figure 4A**). Consistently to the vineyard-wide study, berries contained the lowest concentration of rotundone at both phenological stages (E-L 35 veraison: 7.0 ± 0.8 ng/kg; E-L 38 harvest: 57.5 ± 6.5 ng/kg) compared to non-grape tissues. Also for the first time, rotundone was detected in canes (E-L 35: 65.0 ± 2.4 ng/kg; E-L 38: 106.8 ± 4.0 ng/kg), which represented the first type of lignified grapevine tissue described containing rotundone.

The rotundone content in each grapevine material per shoot was calculated together with the total rotundone content per shoot (**Figure 4B**). The total rotundone content per shoot significantly increased from veraison (16.5 ng per shoot) to harvest (28.8 ng per shoot), and was mainly contributed by the significantly increase of rotundone content in berries (veraison: 0.8 ng per shoot; harvest: 6.5 ng per shoot; *p* < 0.05). The total content of rotundone from leaves, petioles, and peduncles/rachises was significantly higher (*p* < 0.05) than that of berries at both veraison (17.5-folds) and harvest (2.8-folds; **Figure 4B**). The rotundone content in these non-grape tissues could be especially important for wineries conducting machine harvest and whole-bunch fermentation, where significant amount of peduncles/rachises, leaves, and petioles were involved in the fermentation process as previously described, contributing to higher concentration of rotundone in resulted wine.

### Assessment of Rotundone Transportation with Stable Isotope Feeding

**FIGURE 5:**
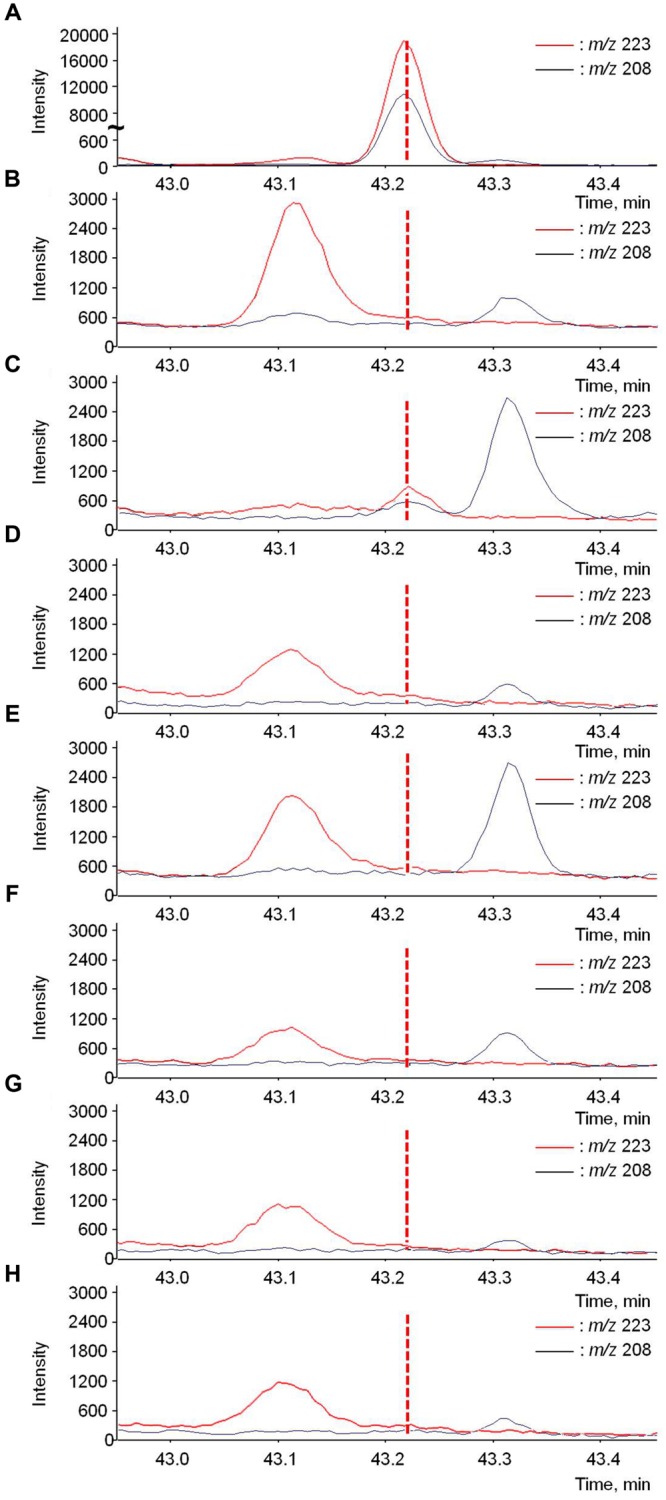
**GC-MS chromatograms of d_5_-rotundone (*m/z* 208 and 223) present in **(A)** positive control, **(B)** negative control, **(C)** treated leaves, **(D)** treated petioles, **(E)** untreated leaves, **(F)** untreated petioles, **(G)** grape berries, and **(H)** grape peduncles/rachises sample.** The positive control graph **(A)** used a different scale of intensity compared to the rest graphs. The peaks near 43.1 and 43.3 min are irrelevant to this study.

Rotundone translocation was firstly assessed by applying stable isotope to grape leaves. d_5_-Rotundone was added to the positive control samples during SPME-GC-MS analysis to locate the compound after treatment. d_5_-Rotundone was observed at the retention time (RT) 43.22 min (**Figure 5A**). As expected, there were no peaks were observed in the negative control sample at the same RT (**Figure 5B**). Furthermore, there were no other compounds in grapevines with peaks of *m/z* 208 and 223 at similar RT, and *m/z* 208 and 223 are valid indicators of d_5_-rotundone as suggested previously (Siebert et al., 2008). In all samples, two small peaks near times 43.12 and 43.31 min represented other compounds irrelevant to the current study, as their peaks were of different RT compared to d_5_-rotundone (**Figure 5**). Small peaks corresponding to d_5_-rotundone were observed in treated leaves (**Figure 5C**), but not in the treated petioles (**Figure 5D**). The d_5_-rotundone detected in the treated leaf samples may correspond to d_5_-rotundone diffusion into the leaf tissues as the residual d_5_-rotundone solution used for leaf treatment has been thoroughly washed off. No d_5_-rotundone was detected in untreated parts of the grapevine (**Figures 5E–H**), showing that d_5_-rotundone translocation in the grapevine did not occur under the experimental conditions described here. Since only very small peaks corresponding to d_5_-rotundone were detected in leaves in direct contact with d_5_-rotundone solution (**Figure 5C**), it is possible that d_5_-rotundone absorbed was not sufficient. Therefore, leaves were cut to facilitate d_5_-rotundone uptake in phloem extraction experiments.

**FIGURE 6:**
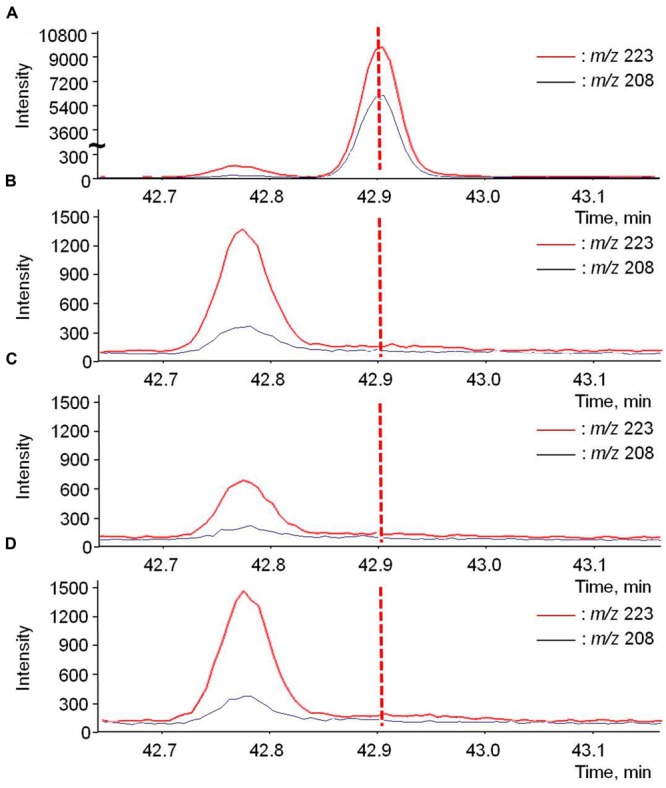
**GC-MS chromatograms of d5-rotundone (*m/z* 208 and 223) present in the phloem exudates of **(A)** positive control-phloem, **(B)** negative control-phloem, **(C)** whole leaves treatment, and **(D)** cut leaves treatment.** The positive control-phloem graph **(A)** used a different scale of intensity compared to the rest graphs. The peak at 42.78 is irrelevant to this study.

The most likely pathway for rotundone to be translocated, via phloem, was assessed by direct extraction of phloem exudate. d_5_-Rotundone was intentionally added to the phloem exudate positive control samples, which exhibited an abundant peak at RT 42.9 min (**Figure 6A**). The RT was slightly different from experiment B1, as the samples were analyzed on SPME-GC-MS separately. As expected, no peak was observed in the negative control at the same RT (**Figure 6B**). The peak at time 42.78 min represented another compound irrelevant to this study, as the peak was of a different RT compared to d_5_-rotundone. Furthermore, no significant peaks corresponding to d_5_-rotundone were observed in the phloem exudates samples of both isotope treatments using whole (**Figure 6C**) and cut leaves (**Figure 6D**). For phloem translocation, it is essential that the compound is able to enter the companion cells and sieve elements from the source tissue and exit at the sink tissue, and this process may involve the passive symplastic pathway or the active apoplasmic pathway (Turgeon and Medville, 2004; Turgeon and Wolf, 2009). The symplastic transport is limited by the size of the molecule, and active apoplasmic transport may be necessary for a secondary metabolite like rotundone (Turgeon and Wolf, 2009). Grapevines have not been reported to present the latter mechanism for sesquiterpenes, and therefore, rotundone translocation via phloem may not be possible in Shiraz grapevines or the phenomenon was not detectable by the techniques used in this study.

**FIGURE 7:**
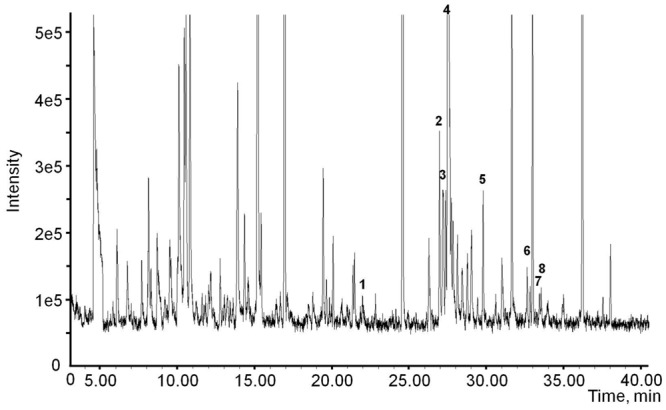
**Chromatograms showing the terpenoids in phloem sap extracted from the petiole: **(1)** geraniol; **(2)** clovene; **(3)** α-ylangene; **(4)** α-copaene (internal standard); **(5)** β-copaene; **(6)** α-muurole ne; **(7)** δ-cadinene; **(8)***cis/trans*-calamenene**.

It is possible that the negative results shown in the isotope feeding experiments is due to low diffusion of d_5_-rotundone into treated leaves. To test this, endogenous rotundone was measured in whole leaves, whole petioles and petiole phloem exudates of the potted vines. Rotundone was detected in the petioles tissues (25.0 ± 4.1 ng/kg), but not in petiole phloem exudates, suggesting that rotundone is produced in the petiole tissues, but not transported in the phloem sap. These results further suggest that rotundone is not likely to be translocated via phloem, since rotundone was also detectable at high concentrations in the leaves (225.2 ± 7.2 ng/kg). The sesquiterpene composition of phloem exudates was further investigated (**Figure 7**). Neither rotundone nor its precursor α-guaiene was detected, while one monoterpenoid, namely geraniol, and six sesquiterpenes (clovene, α-ylangene, β-copaene, α-muurolene, δ-cadinene, and *cis/trans*-calamenene) were observed. Most of the sesquiterpenes detected in this study (except cloven) are products of germacrene D, which is biosynthetically related to a large group of plant sesquiterpenes (Bülow and König, 2000; Zhang et al., 2016). The sesquiterpenes detected may be biosynthesised by phloem cells as shown previously (Hampel et al., 2005) or translocated from other grapevine tissues, such as leaves. The phloem exudate obtained using EDTA extraction method may contain non-mobile materials from the petiole tissue, as EDTA may soften tissues to release metabolites (Turgeon and Wolf, 2009). However, the method used in the current study placed the petioles in completely darkness during extraction, which largely reduces the chance of EDTA damage (King and Zeevaart, 1974). Detailed analysis of the origin of terpene compounds in EDTA phloem exudate requires further investigation.

### Assessment of Local Rotundone Production Induced by Herbivore, Phytohormones and Physical Wounding

**FIGURE 8:**
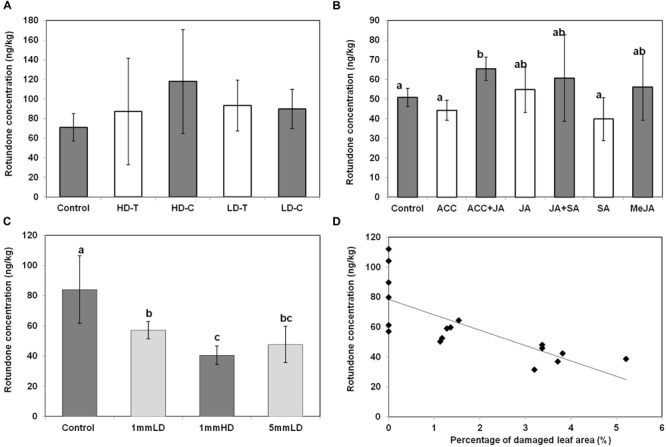
**Influence of herbivore and mimic herbivore attack on the concentration of rotundone in Shiraz grape leaves.** Comparison of rotundone concentration in grape leaves among **(A)** light brown apple moth larvae treatment groups; **(B)** plant hormones treatment groups; **(C)** physical wounding treatment groups. **(D)** The relationship between rotundone concentration in leaves and the percentage of leaf area damage. The equation for the trend line is *y* = 10.26*x* + 78.38 (*R*^2^ = 0.58, RMSE = 15.59, *p* = 0.0004).

Local production of rotundone in grapevine leaves was further investigated using herbivore, physical wounding and phytohormones (**Figure 8**). Large variations in leaves rotundone concentration were observed among herbivore treatments. As a result, no significant differences could be established amongst herbivore treatments and controls (**Figure 8A**). Nevertheless, treated leaves that had been attacked by light brown apple moth larvae showed a tendency to have higher mean rotundone concentrations (95.6 ± 38.0 ng/kg) compared to control samples (71.1 ± 13.9). No significant differences in rotundone concentration were observed between damaged and undamaged sides of leaves in both LD and HD herbivore treatments. Even though terpenoids are major secondary metabolites induced by herbivore attacks in other plant species (Arimura et al., 2005; Howe and Jander, 2008), rotundone may not be the major terpenoids produced as a consequence of feeding by light brown apple moth larvae in *V. vinifera*. A large number of compounds exist in the terpenes family with their universal C5 precursors, isopentenyl pyrophosphate and dimethylallyl pyrophosphate (Dunlevy et al., 2009; Zhang et al., 2016). In an herbivore-induced event like this study, downstream herbivore-induced terpenes may compete with each other for the resource, and rotundone may not outstand in this competition process.

This study tried to mimic two impacts of herbivore influences separately. Phytohormone was applied to plants to simulate oral elicitors. Slight increases in leaf rotundone concentration were only observed in the treatment applying ACC and JA together, but not any other phytohormone treatment groups (**Figure 8B**). This may be explained by the known synergistic effect of ACC and JA signaling, which has been shown to induce higher expression of genes involved in plant herbivore defenses (Bari and Jones, 2009). A previous study showed that conjugate of JA and ACC could up-regulate the production of sesquiterpenes in leaves of *Polygonum longisetum* (Tamogami et al., 2007). However, the sesquiterpenes produced were all originated from precursors rather than germacrene A, where rotundone is derived from Zhang et al. (2016). Furthermore, application of JA and MeJA individually did not significantly alter leaf rotundone concentrations, consistent with a previous report in field-grown *V. vinifera* L. cv. Duras (Geffroy et al., 2014). The SA signaling pathway is different from that of ACC and JA in regulating herbivore-induced terpene synthesis (Pieterse et al., 2012). A previous study in grape cell cultures showed that SA and JA may be antagonistic (D’Onofrio et al., 2009). Although, SA has been reported responsible for regulating the production of some sesquiterpenoids in other plants (Malarz et al., 2007), exogenous SA does not significantly alter leaf rotundone concentrations in grapevine (**Figure 8B**). Our results showed that rotundone biosynthesis could be slightly up-regulated by the conjugative effect of ACC and JA, but not via other signaling pathways.

Intermittent wounding was applied to grapevine leaves to simulate physical damage by herbivores. All three physical wounding conditions were associated with lower leaf rotundone concentrations, with high-density wounding resulting in the lowest rotundone concentration (**Figure 8C**). In addition, the percentage of damaged leaf area was negatively correlated to leaf rotundone concentrations (**Figure 8D**). There are two possible explanations for these observations: (i) mechanical wounding might induce the biosynthesis of other terpenoids at the expense of rotundone (Mithöfer et al., 2005; Bricchi et al., 2010); (ii) mechanical damage to the leaves could increase emission of rotundone and its precursor α-guaiene to the surrounding environment. As a result, the concentration of rotundone left in the leaves may not necessarily increase. Overall, rotundone concentrations in grapevine leaves can be regulated by physical wounding and herbivore-associated elicitors at a limited extend. Physical wounding might reduce rotundone concentrations in grapevine leaves, while conjugates of ACC and JA elicitors can increase it. As a result, the rotundone concentration in leaves could be under complex regulatory control and may not necessarily be modified by herbivores, explaining the observed results of herbivore treatments (**Figure 8A**).

## Conclusion

In this study, we investigated rotundone distribution amongst different grapevine tissues, and confirmed that non-grape tissues contain significantly higher concentration and total amounts of rotundone than the berries. Rotundone was firstly observed in grapevine petioles, peduncles/rachises and lignified cane. This showed that non-grape tissues can be a major source of rotundone, which is of particular interest for winemakers conducting whole-bunch fermentation to maximize this aroma compound in wine. This study further examined the most likely rotundone transportation mechanism from higher concentration vegetative tissues to berries, via phloem, and confirmed that phloem-mediated translocation of rotundone was unlikely under these study conditions. Phloem exudate analysis of sesquiterpenes further excluded the possibility that its precursor, α-guaiene, was translocated in the phloem. For the first time, this study detected geraniol, cloven, and five germacrene D-derived sesquiterpenes in phloem exudates, indicating the possible translocation of these compounds via the phloem or the likely biosynthesis of these compounds in petiole tissues, such as phloem cells, which warrants further investigations. Local productions of rotundone in grapevine leaves can be affected by herbivore activity and mimic treatments including phytochemicals and physical wounding, but to a limited extend. Two aspects of herbivore influences, physical damage and chemical elicitors, had opposing effects on rotundone concentrations in grapevine leaves. As a result, the overall influence of herbivores on leaf rotundone concentrations is uncertain. It is possible that rotundone is a member of herbivore-induced terpenes, but not the major one. Overall, rotundone is widely distributed and produced independently in different tissues of grapevine, interaction between different tissues on its production is unlikely.

## Author Contributions

PZ, SF, EB, MK, MH, and KH conceived and designed research. PZ, YW, and RD conducted experiments. PZ and MH contributed analytical tools. PZ, KH, YW, RD, and SF analyzed data. PZ and KH wrote the manuscript. All authors read and approved the manuscript.

## Conflict of Interest Statement

The authors have the following interests: The field work in this study was supported by a commercial company (Rathbone Wine Group) and the research body of the Australian Wine Research Institute. Co-authors Mark Krstic and Markus Herderich are employed by Australian Wine Research Institute. There are no patents, products in development or marketed products to declare. This does not alter the authors’ adherence to all the Frontiers in Plant Science policies on sharing data and materials.

The reviewer ED declared a shared affiliation, though no other collaboration, with one of the authors KW to the handling Editor DW, who ensured that the process nevertheless met the standards of a fair and objective review.
