# Supplementary material for: Distribution of Rotundone and Possible Translocation of Related Compounds Amongst Grapevine Tissues in Vitis vinifera L. cv. Shiraz
Source: Front Plant Sci. 2016 Jun 21;7:859. doi: 10.3389/fpls.2016.00859 (PMC4914589; doi:10.3389/fpls.2016.00859)
Supplement: Supplementary file 1 [file Table_1.DOCX]

***Supplementary Material***

**Distribution of Rotundone amongst Grapevine Tissues in *Vitis vinifera* L. cv. Shiraz**

Pangzhen Zhang^1^, Sigfredo Fuentes^1^, Yueying Wang^1^, Rui Deng^1^, Mark Krstic^2^, Markus Herderich^3^, Edward W.R. Barlow^1^and Kate Howell^1, *^

^*^ Corresponding author: Tel: +61 3 90353119;

Email address: [khowell@unimelb.edu.au](mailto:khowell@unimelb.edu.au)

**Supplementary Table 1.** Weather record of the vineyard in 2012-13 and 2013-14 seasons (Data from Ararat Prison weather station, Australian Bureau of Meteorology Station No. 089085 – located 15.5kms NW to the experimental site). The date of 80% veraison is taken at 19^th^ Feb 2013 and 28^th^ Feb 2014 and commercial harvest begins on 10^th^ Apr 2013 and 8^th^ Apr 2014.

| **Weather record** | **2012-13** | **2013-14** |
| --- | --- | --- |
| **Mean January Maximum Temperature** | 28.8°C | 30.0°C |
| **Mean January Minimum Temperature** | 11.1°C | 13.4°C |
| **Mean January Temperature** | 20.0°C | 21.7°C |
| **Mean January Solar Exposure** | 29.1 MJ/m^2^ | 27.0 MJ/m^2^ |
| **Mean Maximum Temperature**  **80% veraison to harvest** | 26.3°C | 24.7°C |
| **Mean Minimum Temperature**  **80% veraison to harvest** | 11.5°C | 10.1°C |
| **Mean Temperature**  **80% veraison to harvest** | 18.9°C | 17.4°C |
| **Mean Solar Exposure**  **80% veraison to harvest** | 18.1 MJ/m^2^ | 15.4 MJ/m^2^ |
| **Total rainfall from Oct to Harvest** | 124.1 millimeters | 140.9 millimeters |
| **Total irrigation From Oct to Harvest** | 84.3 millimeters | 60.8 millimeters |
| **Total water precipitation (October to Harvest)** | 208.4 millimeters | 201.7 millimeters |
